# Glycogenic Hepatopathy as the Etiology of Abnormal Liver Chemistries in an Uncontrolled Type I Diabetic Patient

**DOI:** 10.7759/cureus.19755

**Published:** 2021-11-19

**Authors:** Abdullah S Shaikh, Jenine Zaibaq-Krill, Heather L Stevenson, Shehzad Merwat, Sheharyar Merwat

**Affiliations:** 1 Internal Medicine, University of Texas Medical Branch League City Campus, League City, USA; 2 Gastroenterology and Hepatology, Yale School of Medicine, New Haven, USA; 3 Pathology, University of Texas Medical Branch at Galveston, Galveston, USA; 4 Gastroenterology and Hepatology, University of Texas Medical Branch at Galveston, Galveston, USA

**Keywords:** adolescent diabetes, elevated liver transaminases, abnormal liver chemistries, type i diabetes mellitus, glycogenic hepatopathy

## Abstract

Non-alcoholic fatty liver disease (NAFLD) is the most common chronic liver disease in diabetics. However, it is not the sole cause of chronic liver disease in diabetics. We present a case of an 18-year-old male with poorly controlled type I diabetes mellitus who presented for evaluation of asymptomatic elevated liver chemistries. An extensive autoimmune, metabolic, and infectious workup was unrevealing. Liver biopsy was consistent with glycogenic hepatopathy without evidence of steatosis or fibrosis. Increased glycemic control led to his liver enzymes trending down. In conclusion, glycogenic hepatopathy should be considered in poorly controlled type 1 diabetics with elevated liver chemistries.

## Introduction

There is a broad differential diagnosis for hepatomegaly and elevated liver chemistries including hepatitis of viral, autoimmune etiologies, alcoholic and non-alcoholic steatohepatitis (NASH), Wilson disease, and alpha-1 antitrypsin deficiency. Non-alcoholic fatty liver disease (NAFLD) is the most common chronic liver disease in the United States [1] with an incidence of approximately 25% worldwide [2]. It is also the most common chronic liver disease in diabetics with an incidence of 54% [3] but is not the sole cause of liver disease in diabetics. We present a young patient with uncontrolled type 1 diabetes who presented with elevated liver chemistries and was found to have glycogenic hepatopathy.

## Case presentation

An 18-year-old male with poorly controlled type 1 diabetes mellitus (Table 1) and Grave’s disease status post radioactive iodine ablation presented for evaluation of elevated liver chemistries six months after being started on statin therapy. He was started on statin therapy in 2/2019 with a baseline aspartate transaminase (AST) of 78 U/L and alanine transaminase (ALT) of 106 U/L. Prior to this, his liver chemistries were checked in 2013 and were within normal limits. Presenting labs in 8/2019, six months after starting statin therapy, included an ALT of 440 U/L, AST of 479 U/L, and alkaline phosphatase of 221 U/L. Creatinine, calcium, international normalized ratio (INR), bilirubin, protein, albumin, phosphorus, hemoglobin, platelets, and creatinine kinase (CK) were all within normal limits. The patient’s BMI was 26.6 kg/m^2^. His statin was held, and he underwent a significant workup which revealed negative anti-liver-kidney microsomal (LKM) antibody, smooth muscle antibody, antinuclear antibody (ANA), antimitochondrial antibody (AMA), and celiac screen. Alpha-1 antitrypsin, IgG, ceruloplasmin, ferritin, and free T4 levels were all within normal limits (Table 2). Hepatitis IgG was positive as was hepatitis B surface antibody; hepatitis B surface antigen was negative (Table 2). His Hgb A1c was over 14% (Table 1). Liver ultrasound revealed cholelithiasis without cholecystitis, hepatomegaly to 20.7 cm, and fatty infiltration. The only medications the patient was taking were levothyroxine and insulin once the statin was discontinued. He denied tobacco, alcohol, or illicit drug use.

**Table 1:** Lab values. ALT: Alanine transaminase; AST: Aspartate transaminase.

	08/2019	08/2020	11/2020
Lab (Unit)	-	-	-
T Bili (mg/dL)	0.7	-	0.4
T Protein (g/dL)	7.6	-	7.2
Albumin (g/dL)	4.8	-	4.2
ALT (U/L)	440	-	45
AST (U/L)	479	-	75
Alkaline phosphatase (U/L)	221	-	103
Hgb A1c (%)	>14	9.3	-

**Table 2:** Chronic liver disease workup. HBsAb: Hepatitis B surface antibody; HBsAg: Hepatitis B surface antigen; HAV: Hepatitis A virus; LKM: Liver-kidney microsomal antibody; CK: Creatinine kinase; ANA: Antinuclear antibody; AMA: Antimitochondrial antibody.

	08/2019	09/2019	10/2019
Lab (Unit)	-	-	-
CK (U/L)	142	-	-
Free T4 (ng/dL)	-	0.88 (within normal limits)	-
Uric Acid (mg/dL)	-	6.6 (within normal limits)	-
Alpha 1 Anti-trypsin (mg/dL)	-	100 (within normal limits)	-
IgG (mg/dL)	-	896 (within normal limits)	-
Ceruloplasmin (mg/dL)	-	45 (within normal limits)	-
Ferritin (ng/dL)	-	32.3 (within normal limits)	-
Anti-LKM	-	Negative	-
HAV IgM	-	Negative	-
HAV IgG	-	Positive	-
HBsAg	-	Negative	-
HBsAb	-	Positive	-
ANA	-	Negative	-
Anti-Smooth Muscle Ab	-	Negative	-
AMA		Negative	-
Celiac Panel		-	Negative

**Figure 1:**
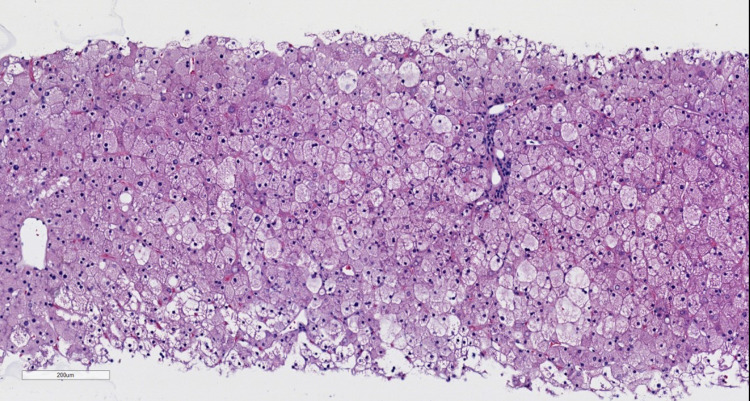
At 4X magnification, the uniformly enlarged, pale hepatocytes are observed.

**Figure 2:**
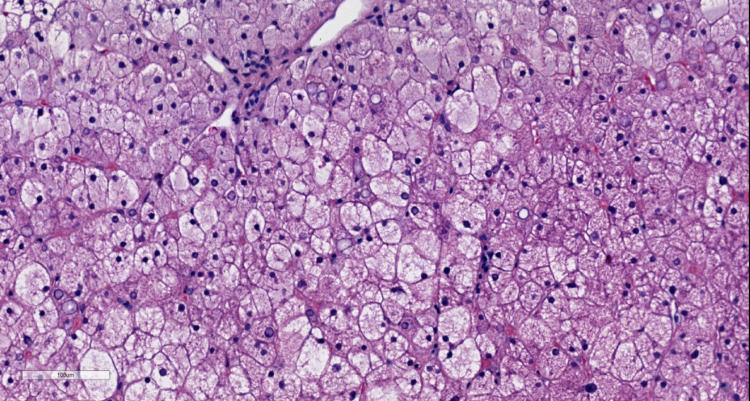
At 10X magnification, hepatocytes with reticulated cytoplasm and prominent cell borders are observed.

**Figure 3:**
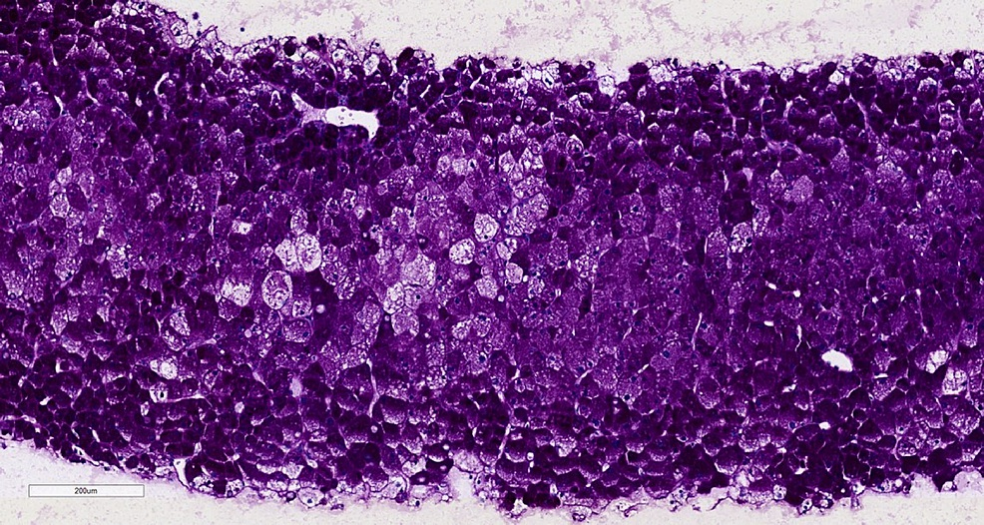
At 10X magnification, a periodic acid-Schiff (PAS) stain highlights abundant intracellular glycogen.

**Figure 4:**
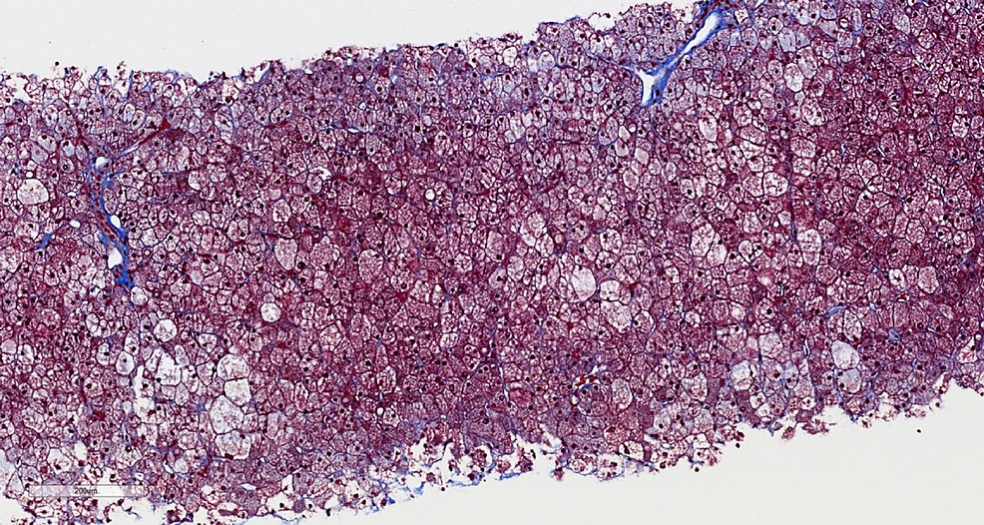
At 4X magnification, a Masson’s trichrome stain shows minimal fibrosis, typical of glycogenic hepatopathy.

The patient remained asymptomatic throughout his workup and never developed abdominal pain, jaundice, ascites, encephalopathy, joint pain, rashes, or pruritis. Due to his negative autoimmune, metabolic, and infectious workup as well as his up-trending liver chemistries (ALT of 424 U/L, AST of 672 U/L, and alkaline phosphatase of 229 U/L with normal albumin, protein, and bilirubin), he underwent a liver biopsy which was consistent with glycogenic hepatopathy without evidence of steatosis or fibrosis (Figures 1-4). He was advised to optimize his glycemic control to assist in resolution of his liver disease. His Hgb A1c decreased to 9.3% over a year with subsequent return of his liver enzymes to levels similar to before his starting of statin therapy (Table 1).

## Discussion

Chronic liver disease encompasses a wide variety of diseases and pathologies but the most common among diabetics is non-alcoholic fatty liver disease (NAFLD) [3]. In type 2 diabetics, insulin resistance appears to be a key pathogenic factor in the development of NAFLD and diabetes [4]. However, in type 1 diabetics, NAFLD must be differentiated from glycogenic hepatopathy which has a much different pathogenesis than NAFLD [5]. Other glycogenic storage diseases were less likely given his normal liver enzymes during childhood and his presentation which was later than when typical pediatric glycogen storage disorders present.

The pathology of glycogenic hepatopathy appears to be related to an increase in glycogen storage in the liver and a decrease in hepatic glycogenolysis due to the presence of both insulin and high levels of blood glucose [6]. This leads to hepatomegaly, elevated liver chemistries, and in some cases, abdominal pain [5]. Liver ultrasound is unable to differentiate glycogenic hepatopathy from NAFLD and requires liver biopsy for definitive diagnosis [4], as was the case for our patient. However, with tight glycemic control, glycogenic hepatopathy and its associated hepatomegaly and elevated liver chemistries often resolve [6], sometimes in as little as four weeks [7]. This contrasts with NAFLD, which requires a combination of weight loss, glycemic control, reduction of triglycerides, and optimization of any other underlying cardiovascular or lifestyle-related risk factors to adequately control. Moreover, glycogenic hepatopathy rarely progresses to fibrosis [8]. In contrast, it is estimated that approximately 33% of patients with NAFLD progress to liver fibrosis over the course of five years [9].

## Conclusions

In conclusion, while NAFLD is the most common liver disease in diabetics, glycogenic hepatopathy should be considered in poorly controlled type 1 diabetics who present with elevated liver chemistries.
